# Pharmacogenomic Drug-Gene Interactions in Geriatric Emergency Department Patients Who Sustained Falls: A Pilot Study

**DOI:** 10.5811/westjem.46553

**Published:** 2025-09-25

**Authors:** Richard D. Shih, Gabriella Engstrom, Abhijit S. Pandya, Gregg B. Fields, Borivoje Furht, Ali A. Danesh, Scott M. Alter, Humberto Munoz, Lisa M. Clayton, Joshua J. Solano, Timothy Buckley, Olivia Hung, Alexander Farag, Mike Wells

**Affiliations:** * Florida Atlantic University, Charles E. Schmidt College of Medicine, Department of Emergency Medicine, Boca Raton, Florida; † Delray Medical Center, Department of Emergency Medicine, Delray Beach, Florida; ‡ Florida Atlantic University, College of Engineering and Computer Science, Department of Electrical Engineering and Computer Science, Boca Raton, Florida; § Florida Atlantic University, Charles E. Schmidt College of Science, Department of Chemistry and Biochemistry, Boca Raton, Florida; || Florida Atlantic University, College of Education, Department of Communication Sciences and Disorders, Boca Raton, Florida; # Bethesda Hospital East, Department of Emergency Medicine, Boynton Beach, Florida

## Abstract

**Introduction:**

Pharmacogenomic-assisted prescribing of medications uses individual genetic information to identify drug-gene interactions. We aimed to assess potential pharmacogenomic drug-gene interactions in geriatric emergency department (ED) patients who sustained a fall.

**Methods:**

This was a prospective study involving 25 older adult ED patients with fall-related injury. Data collected included current medications, demographics, and mechanism of injury. All patients provided a DNA sample, which underwent pharmacogenomic testing by an accredited genetics lab, Each patient’s medications were reviewed against their pharmacogenomic report and categorized as Green (continue to use), Yellow (use with caution) or Red (stop use) based on their genetic information and published interactions by the Clinical Pharmacogenetics Implementation Consortium, Dutch Pharmacogenetics Working Group, and US Food and Drug Administration-approved drug label information. The main study outcome was pharmacogenomic drug-gene interactions.

**Results:**

Of the 25 patients enrolled (median age, 81 years, IQR 76–85), 68% were female. Patients were taking a median of eight medications (IQR 5–11). The most common types were antihypertensives, statins, anticoagulants, and anti-platelet medications. Significant drug-gene interactions (Yellow or Red) were identified in 14/25 patients (56%; 95% CI 35–76%). Further, 6/25 (24%; 95% CI 9–45%) had one or more potentially serious (Red) interactions identified.

**Conclusion:**

We found that in geriatric ED patients with a fall-related injury, most had a significant pharmacogenomic drug-gene interaction. DNA testing identifies these interactions and can assist with pharmacogenomic-guided medication prescribing, which may decrease adverse drug events and improve clinical outcomes.

## INTRODUCTION

As individuals age, they have more medical problems and are prescribed more medications. Polypharmacy, defined by convention as taking more than five medications, has been associated with increased adverse drug events (ADE), hospitalizations and poor outcomes.^1^–^4^ Of these poor outcomes, falling and fall-related injury are among the most important.^1^,^5^ Deprescribing (ie, stopping a medication) is a difficult process.^6^–^8^ Although several methods for medication assessment and possible discontinuation have been published, few have been widely adopted given the many challenges associated with stopping medications.^9^–^13^

Pharmacogenomic tests may identify drug-gene interactions that are responsible for some ADEs. By identifying genetic polymorphisms at high risk for ADE, this may provide a clinician iwith objective information to deprescribe or prescribe medications more precisely and appropriately.^14^,^15^ Authoritative organizations, including the Clinical Pharmacogenetics Implementation Consortium (CPIC) and Dutch Pharmacogenetics Working Group(DPWG), as well as. published clinical guidelines offer guidance on how to use pharmacogenomic results to decrease ADE risk.^16^–^18^

The use of pharmacogenomic prescribing of medications has been reported in pediatrics, psychiatry, oncology and geriatrics.^14^,^15^,^19^,^20^ In the emergency department (ED), several previous studies have shown its potential for use.^21^,^22^ However, these studies focused on one or two cytochrome P450 (CYP) metabolic enzyme systems (CYP2D6 and CYP2C19) or one class of medications (opioids). No previous ED studies have looked at multiple metabolic systems and classes of medications in patients who have fallen.^21^ Our objective in this study was to assess the prevalence of pharmacogenomic drug-gene interactions in geriatric ED patients who suffered a fall. The etiology of falls in geriatric patients is complex and has multifactorial etiologies. This study focuses only on gene-drug interactions that may contribute to falling.

## METHODS

### Study Design and Setting

We conducted an observational cross-sectional study involving 25 patients presenting to a single ED with an annual patient volume of 50,000. The study was approved by the hospital’s affiliated university institutional review board (IRB).

### Selection of Participants

In this pilot study we used a convenience sample of older adults presenting to the ED after a ground-level fall. All eligible patients were approached by trained research assistants (RA) and invited to participate. Study enrollment occurred only during the hours that RAs were present in the ED (9 am to 11 pm daily). Participants in this study represented a subset of patients enrolled in a larger randomized controlled trial on falling in older adults (unrelated to pharmacogenomics). The specific inclusion criteria were patients ≥ 65 years of age who had presented to the ED with a fall-related injury. The criteria for exclusion from the study were failure to obtain consent; patients who had suffered penetrating injuries > 24 hours prior to presentation; patients transferred from another hospital; and patients who were in hospice care or had do not resuscitate status.

**Population Health Research Capsule**

What do we already know about this issue?

*Geriatric emergency department (ED) patients may have drug-gene interactions that contribute to adverse drug events. DNA analysis and use for medication prescribing may prevent these*.

What was the research question?

*What is the prevalence of potential drug-gene interactions in geriatric ED patients who have fallen?*

What was the major finding of the study?

*In this small pilot study, 56% (95% CI 35–76%) were found to have a potentially serious drug-gene interaction*.

How does this improve population health?

*DNA testing can identify potential medication drug-gene interactions. This information may be useful to guide prescribing practices*.

### Data Collection

Identified patients who met the study’s inclusion criteria were approached to complete an IRB-approved informed consent form for study participation, and their data were collected by the RAs. Data collected included current medications (prescribed pre-injury), demographic data, mechanism of injury, past medical history, and ED diagnosis. In addition, patients provided a DNA sample obtained via a single cheek swab sample from inside the patient’s mouth. (The subjects were not allowed to eat, drink, or smoke for 30 minutes prior to swabbing.) We used the pharmacogenomic test MatchMyMeds (DNA Labs, Boca Raton, FL, https://dnalabs.ca), which reports on potential drug-gene interactions for 134 medications. The genetics company is accredited by the Clinical Laboratory Improvement Amendments, the College of American Pathologists, the State of California Department of Public Health, the American Association of Blood Banks, and the New York State Department of Health.

The MatchMyMeds test analyzes for 23 different metabolic enzyme systems and reviews potential drug-gene interactions for 134 different medications (see Supplementary Table). The report is based on the Clinical Pharmacogenetics Implementation Consortium,^16^ the Dutch Pharmacogenetics Working Group,^17^,^18^ and US Food and Drug Administration-approved drug label information to provide detailed drug and dosing recommendations. In addition, based on the individual’s genetics, the report categorized 134 medications as Green (go: use label recommended dosage and administration), Yellow (caution: use with caution and read detailed recommendations for potential dose adjustment), or Red (stop: select alternative treatment if possible and read detailed recommendation for detail). DNA Labs used the MassARRAY System (Agena Bioscience GmbH, Hamburg, Germany), to perform the genotyping.^23^ Copy number variant (CNV) analysis was performed using the Agena Veridose panel, to detect CNVs and hybrid alleles. The lab did not assess drug-drug interactions from medication co-consumption.

### Outcomes

The main study outcome was to identify pharmacogenomic drug-gene Interactions. Yellow and Red medications were defined as serious interactions. A secondary outcome was to assess whether a medication with a significant gene-drug interaction was also identified in the Beers Criteria, which lists potentially inappropriate medications for older adults.^24^

### Analysis

We conducted descriptive analyses to assess the prevalence and severity of drug-gene interactions, as well as to identify the specific drugs affected. We quantified the frequency of interactions within each risk category (Green, Yellow, and Red) and characterized the severity of these interactions based on their potential impact on patient safety and treatment efficacy.

## RESULTS

We enrolled 25 patients. The median age was 81 years (IQR 76–85), and 68% were female. The most frequent co-morbidities were hypertension (64%), atrial fibrillation (32%), coronary artery disease (28%) and stroke (8%). All patients were on medications for pre-existing co-morbidities (see Table 1). Patients were taking a median of eight medications (IQR 5–11). The most common types were antihypertensives, statins, anticoagulants, and anti-platelet medications. All 25 patients had suffered a fall, as this was part of the criteria for inclusion in the study. Most had contusions, fractures, and wounds. Several also had significant medical problems such as stroke, head injury, and syncope.

**Figure 1:**
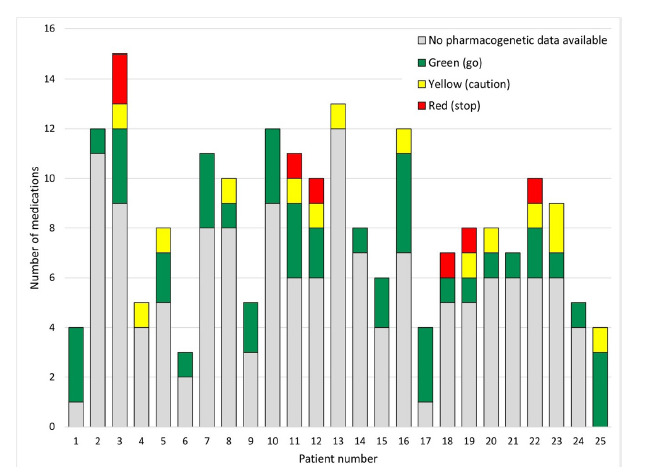
Possible drug-gene medication interactions for each patient.

When considering the pharmacogenomic data and drug-gene interactions, of our 25 patients 14 (56%, 95% CI 35–76%) had one or more Yellow or Red medications (see Figure 1) for a total of 20 significant gene-drug interactions (see Table 2). Further, 6/25 (24%, 95% CI 9–45%) had one or more Red medications identified (Figure 1). The implicated medications were those that could increase risk of falling with several drugs with the potential for increased sedation (escitalopram, tramadol, trazodone, oxycodone, and duloxetine) and others potentially causing bradycardia or hypotension (metoprolol and amlodipine).

## DISCUSSION

As individuals get older, they develop more medical problems and are prescribed more medicines. The more medications that someone takes, the higher the risk for an ADE.^25^ Polypharmacy—taking five or more medicines—is a major risk factor for an ADE.^26^ One common ADE is a fall and fall-related injuries. These occur commonly, with approximately 30% of older adults sustaining a fall each year.^27^ Although a medication- related ADE can cause falls, there are other etiologic factors that contribute. Our study focused only on gene-drug interactions that may contribute to falling.

Shaver et al in 2021 showed that mortality risk from falls in older persons in the US increased steadily from 1999 to 2017, as the use of fall-risk increasing drugs rose. During that period, the percentage of older people taking a fa-risk increasing drug increased from 57% to 97%.^3^ On average, 750 older adults in the US are hospitalized each day due to an ADE, and 50% are taking ≥ seven medications.^5^,^13^ Further, it is estimated that 60% of older people take one or more medicines that are unnecessary.^28^ Among geriatric ED patients about 75% are prescribed one or more medication classified as “avoid” or “use with caution.”^6^ However, stopping a medication (deprescribing) has proven to be difficult for patients and physicians.^13^

Although various methods for medication assessment and possible discontinuation have been published, most have not been widely adopted.^9^–^11^,^24^,^29^ Deprescribing presents several significant challenges in clinical practice including physicians who lack training in stopping medications or are concerned about worsening symptoms; patients who worry about worsening symptoms; concern regarding withdrawal and the time required to discuss medication changes; and patients who feel their doctor is “giving up” and is no longer treating their disease.^13^,^16^,^30^

Pharmacogenomics, a genetics-based method to assess for possible risk of ADEs, could one day become standard information for physicians to help provide more precise and personalized prescribing of medications. Pharmacogenomics involves gene testing to assess how an individual metabolizes medications. Hepatic enzymes in the P450 systems (ie, CYPD6, CYP3A4, and CYP2C19) are responsible for metabolizing many commonly used medications such as atorvastatin, diazepam, warfarin, rivaroxaban, sertraline, and many others). In addition, CYP2D6 has many different genetic polymorphisms and, depending on the inherited alleles, the individual CYP2D6 system may function as a poor, normal or ultra-rapid metabolizer.^3^ Most people are normal metabolizers; and in our current “one size fits all” approach to dosing medications, the standard dose will generally provide concentrations in the therapeutic window predictably. However, if the individual is a poor metabolizer or an ultra-rapid metabolizer, a given medication may be in a toxic or ineffective concentration range due to the gene-drug interaction.

Previous studies suggest that most individuals have one or more gene phenotypes that can cause an adverse drug event with commonly prescribed medications.^32^–^34^ In a study of close to eight million Veterans Administration patients, based on genetic allele frequency and medication history, it was estimated that most (> 90%) of the individuals had at least one actionable pharmacogenomic variant.^34^

In our study each patient’s medications was assessed for a potential drug-gene interaction based on DNA Labs’ pharmacogenomic report. Of our 25 patients there were 20 (Red or Yellow) significant potential drug-gene interactions identified, with 56% taking one or more “Yellow” or “Red” medications (see Figure and Table 2). In addition, 20% had at least one “Red” drug-gene medication interaction identified. Examples of significant gene-drug interactions include the following:

- Reduced CYP2D6 enzyme activity in someone taking metoprolol. These individuals are poor metabolizers of metoprolol and have a three-to-tenfold higher concentration compared to a normal metabolizer. Further, poor metabolizers had a five times higher rate of ADE compared to non-poor metabolizers. These ADE include symptomatic bradycardia and dizziness.^35^
- Reduced CYP3A4 enzyme activity in someone taking trazodone may lead to changes in concentrations of the parent compound and active metabolites.^36^,^37^ This may lead to increased sedation, dizziness and falls.”^36^–^38^

An additional important finding is that many of the gene-drug interactions identified are not on the American Geriatrics Society 2023 updated Beers Criteria (see Table 4).^24^ The Beers Criteria is a list of potentially inappropriate medications for older adults, which was developed by a panel of interprofessional experts who reviewed the published literature to establish this list. However, the medications involved in 11 of the 20 (55%) significant potential drug-gene interactions identified in our study are not on the Beers list (whose methodology does not attempt to identify drug-gene interactions).

An important question is whether drug-gene interactions have a causal link with falling. It was not an objective in this study to evaluate potential causality, but this is a critical issue to consider. It has been well established that multiple factors contribute to a risk of falling. It has also been well established that medications can be significant risk factors. Further, there is good preliminary evidence that there is an association between the occurrence of falls in older adults and the metabolic capacity of certain enzyme systems (detectable through pharmacogenomic analysis).^39^ The interactions between the risk factors for falling have not been fully elucidated, nor has single-factor causality been clearly determined for any factor. However, medication optimization is one of the most easily addressed risk factors and, therefore, worthy of attention. Clearly, other factors may compound the effect of suboptimal medication therapy. In addition, not all drugs are likely to impart the same degree of risk or type of risk. In the end, however—irrespective of the case for causality related to falls—a strong consensus is emerging that personalized drug therapy is essential in older adults.^40^

Technological advances over the past several decades have drastically increased the speed of analyzing DNA samples and decreased the costs. (As of this writing, the consumer cost for DNA Labs MatchMyMeds testing is $275).^41^ While the results of pharmacogenomic testing performed in the ED are not yet available in real time, it is likely that pharmacogenomic data will be routinely integrated into the electronic health record. However, to be practically useful, this information would need to be accompanied by clinical decision-support alerts that would identify gene-drug interactions and how they should be addressed, at the point of prescribing. This could be an especially valuable tool when managing high-risk medications in patients at risk of falling.

When this pharmacogenomic information is already available, such as from previous outpatient testing, emergency physicians could use the data to identify medications that may be inappropriate or pose elevated risk, adjust or discontinue certain prescriptions to lower fall risk, and communicate relevant pharmacogenomic concerns to the patient’s primary care physician for ongoing management. Incorporating genetic testing into routine care would allow clinicians to personalize drug therapy, reduce the likelihood of adverse drug reactions, and improve treatment outcomes for older adults. There is convincing evidence that about half of all older adults are prescribed at least one pharmacogenomically actionable drug, and between 25–35% receive two or more such drugs. These findings indicate that exposure to multiple medications with drug-gene interactions is common, supporting the potential value of implementing broad, pre-emptive genomic testing.^42^ There is no reason this could not be introduced into the ED, as this is particularly relevant in older adults at risk of adverse drug events, even if not yet routine practice. This would address one of the core obligations of physicians to “first do no harm.”

The potential future utility of pharmacogenomics in emergency medicine is considerable, particularly as national healthcare systems move toward integrating genomic data into routine care. For example, the United Kingdom’s National Health Service (NHS)—citing evidence that it is possible to reduce adverse drug reactions and unplanned hospitalizations among older adults—has committed to embedding pharmacogenomic testing into clinical practice; NHS pilot programs focused on high-risk conditions are ongoing. While not yet ED-specific, the availability of pharmacogenomic data at the point of care could allow emergency physicians to tailor prescribing and deprescribing decisions more safely and effectively. As pharmacogenomic-guided therapy becomes standard over the next decade, leaders in emergency medicine will need to address the associated challenges associated with this therapy, especially in terms of time demands. Integrating pharmacogenomic alerts into the EHR, potentially supported by clinical decision-support tools driven by artificial intelligence, may offer a practical and efficient way to enhance prescribing without increasing clinician burden. Nevertheless, further research is essential to evaluate the real-world impact of pharmacogenomic optimization in emergency care. Given the growing importance of precision medicine, this research should be a priority.

## LIMITATIONS

This study enrolled a convenience sample of 25 patients without a control group. Although the sample was small, it was a subset of a larger study with the population characteristics of interest to us. Our main objective in this pilot study was to identify the real-world scope of the potential problem (drug-gene interactions) rather than provide an accurate population estimate of its prevalence. Despite the small sample size, the 95% CI revealed that even the lower limit of drug-gene interaction prevalence (35%) would constitute a clinically significant proportion of older adult patients. Since other studies have estimated up to a 90% theoretical prevalence of drug-gene interactions in older adults, this study, despite its limitations in generalizability. provides important confirmatory evidence. However, a larger study at multiple centers would likely provide more accurate estimates of the incidence and types of these interactions.

A second limitation is that the gene-drug interactions identified are potential, and ADEs were not examined as part of this study. Further, some of the drug-gene interactions that we identified may not cause significant ADEs and may be the reason that these drugs do not appear on the Beers list. Further, pharmacogenomics may identify drugs that are on the Beers list and may be useful to categorize the risk of harm of drugs on the list, in patients with pharmacogenomically detected vulnerability. A randomized controlled trial using the pharmacogenomic-assisted prescribing of medications would be the ideal method to assess for ADEs and the clinical impact of the significant gene-drug interactions that were identified.

A third limitation is that even if individual pharmacogenomic data were available, it is not clear whether physicians would use the data for improved prescribing. One of the barriers is the difficulty in interpretating the pharmacogenomic data, leading to an increased amount of time and lack of user-friendly guideline information.^16^,^30^,^42^ We did not assess this factor; it is another future area of research for the implementation of pharmacogenomic-assisted prescribing practices.

Another limitation is that our study only assessed gene-drug interactions. Several other factors play a role in how cytochrome p450 metabolism of medications occurs. Medications, foods, and medical conditions can augment or inhibit drug metabolism. An example of a common medication interaction is the CYP3A4 inhibitory effect of diltiazem. When co-administered with direct oral anticoagulants their metabolism may be inhibited, and a higher bleeding risk has been seen in these patients.^44^ Our study did not investigate these other factors and did not assess their effect on the gene-drug interactions reported.

Lastly, the patients’ use of medication was assessed by self-report. We did not assess drug levels or other confirmation of medication compliance. (For example, patients reporting medications that they were not actually taking would not represent true gene-drug interactions.)

## CONCLUSION

In this pilot study our aim was to estimate the prevalence of pharmacogenomic drug-gene interactions in geriatric ED patients who had presented for a fall-related injury. We found that nearly 60% of these patients had significant pharmacogenomic drug-gene interactions with potential clinical impact. We did not attempt to establish a causal relationship between these interactions and the patients’ falls. Nonetheless, given the multifactorial nature of the risk of falling, these findings suggest that pharmacogenomic-guided medication prescribing (or deprescribing), could have the potential to decrease adverse drug effects and thereby reduce the negative impact of medications on the risk of falling. In the near future the results of DNA testing might be available in the electronic health record, which would make this possible, even in the ED.

## Supplementary Information



## Figures and Tables

**Table 1:** Clinical characteristics of elderly patients with pre-existing morbidities enrolled in a study of drug-gene interactions and increased risk of falling.

	Patients N = 25 (%)
Past Medical History, n (%)	
Hypertension	16 (64)
Atrial fibrillation	8 (32)
Coronary artery disease	7 (28)
Solid tumor/cancer active	4 (16)
Cerebrovascular attack	2 (8)
Valve replacement	2 (8)
Myocardial infarction	2 (8)
Diabetes	1 (4)
Chronic obstructive pulmonary disease	1 (4)
Congestive heart failure	1 (4)
Pulmonary embolism	1 (4)
Peripheral vascular disease/peripheral stent	1 (4)
Chronic kidney disease	1 (4)

**Table 2:** Significant drug-gene interactions.

Pt	Drug	Enzyme system involved	Flag	Possible clinical significance	Action recommended	Appears in American Geriatric Society Beers Criteria list^15^
3	Escitalopram	Reduced CYP2C19 activity	Yellow	Serum levels increased	Be alert for drug-related adverse effects, especially with co-administration of other drugs that inhibit CYP2C19 enzyme activity.	No
	Metoprolol	Reduced CYP2D6 activity	Red	Serum levels increased – hypotension, bradycardia	Select alternative drug.	No
	Clopidogrel	Reduced CYP2C19 activity	Red	Serum levels decreased – increased risk of thrombosis	Select alternative drug.	No
**4**	Pantoprazole	Reduced CYP2C19 activity	Yellow	Serum levels increased	Be extra alert for lack of efficacy and adverse effects, especially with co-administration of other drugs that inhibit CYP2C19 or CYP3A4 enzyme activity.	Yes
**5**	Tramadol	Elevated CYP2D6 activity	Yellow	Increased level of metabolite (morphine)	Reduce dose by 30% and be alert to adverse drug events.	Yes
**8**	Paroxetine	Reduced CYP2D6 activity	Yellow	Serum levels increased	Be extra alert, especially with co-administration of other drugs that inhibit CYP2D6 enzyme activity.	Yes
**11**	Warfarin	CYP2C9 and VKORC1 variants	Yellow	Variable effect on serum levels	The genetic information below can be entered into the warfarindosing.org form to estimate the most appropriate therapeutic dose in patients new to warfarin.	Yes
	Metoprolol	Reduced CYP2D6 activity	Red	Serum levels increased – hypotension, bradycardia	Select alternative drug (eg, bisoprolol, carvedilol) or reduce dose by 50%.	No
**12**	Escitalopram	Reduced CYP2C19 activity	Yellow	Serum levels increased	Be alert for drug-related adverse effects, especially with co-administration of other drugs that inhibit CYP2C19 enzyme activity.	No
	Clopidogrel	Reduced CYP2C19 activity	Red	Serum levels decreased – increased risk of thrombosis	Avoid standard dose (75 mg) clopidogrel if possible. Use prasugrel or ticagrelor at standard dose if no contraindication.	No
**13**	Pantoprazole	Reduced CYP2C19 activity	Yellow	Serum levels increased	Dose adjustment is not recommended, but be extra alert for lack of efficacy and adverse effects, especially with co-administration of other drugs that inhibit CYP2C19 or CYP3A4 enzyme activity.	Yes
**16**	Trazodone	Reduced CYP3A4 activity	Yellow	Serum levels increased	A lower dose of trazodone should be considered, especially if co-administered with a CYP3A4 inhibitor.	No
**18**	Metoprolol	Reduced CYP2D6 activity	Red	Serum levels increased – hypotension, bradycardia	Select alternative drug (eg, bisoprolol, carvedilol) or reduce dose by 50%.	No
**19**	Celecoxib	Reduced CYP2C9 activity	Yellow	Serum levels increased	Use lowest effective dose and be extra alert for adverse effects, especially with co-administration of other drugs that inhibit CYP2C9 enzyme activity.	Yes
	Oxycodone	Reduced CYP2D6 activity	Red	Serum levels increased – toxic opioid effect	Select alternate drug—not tramadol or codeine—or be alert to symptoms of insufficient pain relief, especially with co-administration of other drugs that inhibit CYP2D6 enzyme activity.	Yes
**20**	Duloxetine	Reduced CYP2D6 and elevated CYP1A2 activity	Yellow	Serum levels increased	Caution is advised—insufficient evidence to support a dosage adjustment.	Yes
**22**	Pantoprazole	Elevated CYP2C19 activity	Yellow	Serum levels decreased	Consider dose increase by 400% and be extra alert to insufficient response.	Yes
	Escitalopram	Elevated CYP2C19 activity	Red	Serum levels decreased	Consider an alternative drug NOT predominantly metabolized by CYP2C19.	No
**23**	Amlodipine	Reduced CYP3A4 activity	Yellow	Serum levels increased – hypotension	Use label recommended dosage and administration, and be extra alert for side effects including symptoms of hypotension and edema.	No
	Apixaban	Reduced CYP3A4 activity	Yellow	Serum levels increased – increased bleeding	The reduction in CYP3A4 enzyme activity in this case may or may not be sufficient to significantly increase exposure; thus, there is insufficient data to support a dosage adjustment, although a dose reduction to 2.5mg twice daily could be considered.	No
**25**	Simvastatin	Reduced CYP3A4 and CYP2C6 activity	Yellow	Serum levels increased – increased risk of rhabdomyolysis	Prescribe a lower dose or consider an alternative statin.	No

*Yellow*: use with caution; read recommendations for potential dose adjustment. *Red*: select alternative treatment if possible.

The term reduced activity is used to indicate a poor metabolizer phenotype; similarly, elevated activity is used to indicate an ultra-rapid metabolizer phenotype. No data are presented for patients 1, 2, 6, 7, 9, 10, 14, 15, 17, 21, and 24 as they had no significant drug-gene interactions.

*Pt*, patient.
