# Supplementary material for: Pharmacogenomic Drug-Gene Interactions in Geriatric Emergency Department Patients Who Sustained Falls: A Pilot Study
Source: West J Emerg Med. 2025 Sep 25;26(5):1414–22. doi: 10.5811/westjem.46553 (PMC12591660; doi:10.5811/westjem.46553)
Supplement: Supplementary file 1 [file wjem-26-1414-s001.docx]

Supplementary Table. Metabolic Enzyme System Genes Analyzed and Medications Assessed for Drug-Gene Interactions

|  |  |
| --- | --- |

| *Gene Symbol* | *Gene Name* |
| --- | --- |
| *ABCB1* | ATP binding cassette subfamily B member 1 (ABCB1) gene |
| *APOE* | Apolipoprotein E (APOE) gene |
| *COMT* | Catechol-O-methyl-transferase (COMT) gene |
| *CYP1A2* | Cytochrome P450 1A2 (CYP1A2), mixed-function oxidase system gene |
| *CYP2B6* | Cytochrome P450 2B6 (CYP2B6), mixed-function oxidase system gene |
| *CYP2C19* | Cytochrome P450 2C19 (CYP2C19), mixed-function oxidase system gene |
| *CYP2C9* | Cytochrome P450 2C9 (CYP2C9), mixed-function oxidase system gene |
| *CYP2D6* | Cytochrome P450 2D6 (CYP2D6), mixed-function oxidase system gene |
| *CYP3A4* | Cytochrome P450 3A4 (CYP3A4), mixed-function oxidase system gene |
| *CYP3A5* | Cytochrome P450 3A5 (CYP3A5), mixed-function oxidase system gene |
| *DPYD* | Dihydropyrimidine dehydrogenase (DPYD) gene |
| *DRD2* | Dopamine D2 receptor (DRD2) gene |
| *F2* | Coagulation factor II (F2) gene |
| *F5* | Coagulation factor V (F5) gene |
| *GLP1R* | Glucagon-like peptide 1 (GLP1R) gene |
| *MTHFR* | Methylenetetrahydrofolate reductase (MTHFR) gene |
| *NUDT15* | Nudix hydrolase 15 (NUDT15) gene |
| *OPRM1* | Opioid receptor mu 1 (OPRM1) gene |
| *PNPLA5* | Patatin like phospholipase domain containing 5 (PNPLA5) gene |
| *SLCO1B1* | Solute carrier organic anion transporter family member 1B1 (SLCO1B1) gene |
| *SULT4A1* | Sulfotransferase Family 4A Member 1 (SULT4A1) gene |
| *TPMT* | Thiopurine S-methyltransferase (TPMT) gene |
| *VKORC1* | Vitamin K epoxide reductase complex subunit 1 (VKORC1) gene |
| *Class* | ***Drugs*** |
| Anti-Anxiety/Anti-Depression | amitriptyline (Elavil), amoxapine (Asendin), bupropion (Wellbutrin), citalopram (Celexa), clomipramine (Anafranil), desipramine (Desipramine), doxepin (Sinequan, Silenor), duloxetine (Cymbalta), escitalopram (Cipralex), fluvoxamine (Luvox), imipramine (Impril), levomilnacipran (Fetzima), mirtazapine (Remeron), nortriptyline (Aventyl), paroxetine (Paxil), protriptyline (Vivactil), sertraline (Zoloft), trazodone (Oleptro), trimipramine (Trimipramine), venlafaxine (Effexor), vilazodone (Viibryd), vortioxetine (Trintellix), diazepam (Valium) |
| Birth Control | hormonal contraceptives for systemic use |
| Cardiovascular | acenocoumarol (Sintrom), amlodipine (Norvasc), apixaban (Eliquis), atorvastatin (Lipitor), clopidogrel (Plavix), cilostazol (Pletal), dronedarone (Multaq), flecainide (Tambocor), metoprolol (Lopresor, Betaloc), phenprocoumon, propafenone (Propafenone), simvastatin (Zocor), warfarin (Coumadin), fluvastatin (Lescol, Fluvastatin), lovastatin (Advicor, Mevacor), pitavastatin (Livalo, Zypitamag), pravastatin (Pravachol), rosuvastatin (Crestor), ranolazine (Ranexa), rivaroxaban (Xarelto) |
| Dentistry | cevimeline (Evoxac) |
| Dermatology | abrocitinib (Cibinqo) |
| Endocrinology | eliglustat (Cerdelga) |
| Gastroenterology | aprepitant (Emend), dexlansoprazole (Dexilant), dronabinol (Marinol), esomeprazole (Nexium), lansoprazole (Prevacid), metoclopramide (Reglan), omeprazole (Losec), ondansetron (Zofran), pantoprazole (Pantoloc), tropisetron (Navoban), rabeprazole (Aciphex) |
| Immunology | azathioprine (Imuran), dexamethasone (Dexamethasone), tacrolimus (Prograf) |
| Infectious Diseases | efavirenz (Sustiva), voriconazole (Vfend) |
| Neurology | donepezil (Aricept), eszopiclone (Lunesta), fosphenytoin (Cerebyx, Sesquient), phenytoin (Dilantin), clobazam (Frisium), siponimod (Mayzent), tetrabenazine (Nitoman), brivaracetam (Brivlera, Briviact), deutetrabenazine (Austedo), dextromethorphan and Quinidine (Nuedexta), valbenazine (Ingrezza), lacosamide (Vimpat), pitolisant (Wakix), Zopiclone (Imovane) |
| Oncology | mercaptopurine (Purinethol), 5-fluorouracil (Adrucil), capecitabine (Xeloda), tamoxifen (Nolvadex), thioguanine (Lanvis), cabazitaxel (Jevtana), cisplatin (Platinol), tegafur (Teysuno) |
| Pain | almotriptan (Axert), buprenorphine and naloxone (Suboxone), codeine, fentanyl (Duragesic), eletriptan (Relpax), ibuprofen (Advil, Motrin), oxycodone, celecoxib (Celebrex), hydrocodone, lornoxicam (Chlortenoxicam), tenoxicam (Tenoxicam), piroxicam, tramadol (Tridural, Tramacet), zolmitriptan (Zomig) |
| Psychiatry | aripiprazole (Abilify), atomoxetine (Strattera), brexpiprazole (Rexulti), clozapine (Clozaril), guanfacine (Intuniv, Tenex), haloperidol (Haloperidol), lurasidone (Latuda), perphenazine, pimozide (Orap), pimavanserin (Nuplazid), risperidone (Risperdal), thioridazine (Mellaril), zuclopenthixol (Clopixol), amphetamine (Adderall, Adzenys), iloperidone (Fanapt), lisdexamfetamine (Vyvanse), quetiapine (Seroquel) |
| Pulmonology | fluticasone furoate and vilanterol (Breo Ellipta), fluticasone, umeclidinium, and vilanterol (Trelegy Ellipta), umeclidinium and vilanterol (Anoro Ellipta) |
| Rheumatology | carisoprodol (Soma), flurbiprofen, lesinurad (Zurampic), meloxicam (Mobic) |
| Urology | tamsulosin (Flomax), dapoxetine (Priligy), fesoterodine (Toviaz), tolterodine (Detrol), darifenacin (Enablex), sildenafil (Viagra), tadalafil (Cialis) |
